# Predicting anaplastic lymphoma kinase rearrangement status in patients with non-small cell lung cancer using a machine learning algorithm that combines clinical features and CT images

**DOI:** 10.3389/fonc.2022.994285

**Published:** 2022-10-20

**Authors:** Peng Hao, Bo-Yu Deng, Chan-Tao Huang, Jun Xu, Fang Zhou, Zhe-Xing Liu, Wu Zhou, Yi-Kai Xu

**Affiliations:** ^1^ Nanfang Hospital, Southern Medical University, Guangzhou, China; ^2^ School of Medical Information Engineering, Guangzhou University of Chinese Medicine, Guangzhou, China; ^3^ School of Biomedical Engineering, Southern Medical Uinversity, Guangzhou, China

**Keywords:** tomography, X-ray computed, anaplastic lymphoma kinase, gene mutation, non-small cell lung cancer, machine learning, texture

## Abstract

**Purpose:**

To develop an appropriate machine learning model for predicting anaplastic lymphoma kinase (ALK) rearrangement status in non-small cell lung cancer (NSCLC) patients using computed tomography (CT) images and clinical features.

**Method and materials:**

This study included 193 patients with NSCLC (154 in the training cohort, 39 in the validation cohort), 68 of whom tested positive for ALK rearrangements and 125 of whom tested negative. From the nonenhanced CT scans, 157 radiomic characteristics were extracted, and 8 clinical features were collected. Five machine learning (ML) models were assessed to find the best classification model for predicting ALK rearrangement status. A radiomic signature was developed using the least absolute shrinkage and selection operator (LASSO) algorithm. The predictive performance of the models based on radiomic features, clinical features, and their combination was assessed by receiver operating characteristic (ROC) curves.

**Results:**

The support vector machine (SVM) model had the highest AUC of 0.914 for classification. The clinical features model had an AUC=0.805 (95% CI 0.731–0.877) and an AUC=0.735 (95% CI 0.566–0.863) in the training and validation cohorts, respectively. The CT image-based ML model had an AUC=0.953 (95% CI 0.913–1.0) in the training cohort and an AUC=0.890 (95% CI 0.778–0.971) in the validation cohort. For predicting ALK rearrangement status, the ML model based on CT images and clinical features performed better than the model based on only clinical information or CT images, with an AUC of 0.965 (95% CI 0.826–0.882) in the primary cohort and an AUC of 0.914 (95% CI 0.804–0.893) in the validation cohort.

**Conclusion:**

Our findings revealed that ALK rearrangement status could be accurately predicted using an ML-based classification model based on CT images and clinical data.

## Introduction

Lung cancer is the leading cause of cancer-related death worldwide. In 2013, in women aged 40 to 59 years, lung cancer surpassed breast cancer as the main cause of cancer death (1). Non-small cell lung cancer (NSCLC) is the most common histological subtype, accounting for 85%-90% of lung cancers (2). In the past decade, the emergence of novel drugs that target signalling pathways activated by genetic changes, for example, EGFR mutations and ALK rearrangement status, has revolutionized the treatment of NSCLC patients (3). The presence of an ALK rearrangement protein has been discovered in a small percentage of NSCLC patients, mostly in those with lung adenocarcinoma (3). Approximately 5% of lung adenocarcinomas have ALK rearrangement status, which is mutually exclusive with EGFR mutations. Crizotinib is a promising ALK fusion status inhibitor (4). Thus, identifying ALK rearrangements in NSCLC patients is crucial for therapy planning.

Because histologic and genetic information from invasive biopsies is often taken from only a section of a generally heterogeneous tumour, this characterization information does not provide a thorough depiction of functional and physiological aspects of lesions (5). The most common method for diagnosing and assessing treatment response of lung malignancies is computed tomography (CT). Thus, previous research has examined the link between some gene mutations in lung cancer and clinical features and radiological characteristics of lung cancer (6). Some CT imaging features, such as central tumour location, pleural effusion, lobulated margin, large mass and distant metastases, have been linked to ALK gene rearrangements in these studies (7–13). However, the evaluation of these radiological characteristics of lung cancer, is time-consuming and greatly dependent on the radiologist’s knowledge. Machine learning (ML) is a computer-based method for diagnosing lung cancer, predicting survival, and forecasting gene mutations. It can help radiologists discover more about the phenotype of a tumour including that is not obvious on CT scans (14–19). To avoid overfitting and develop robust predictive or prognostic models, a successful radiomic prediction study requires several phases, including accurate statistical analysis, feature selection, and classification. To select a subset of features that can be merged into a multiparametric model, a variety of ML algorithms can be utilized. Although radiomic analysis has used a variety of ML approaches for categorization, there is no “one size fits all” solution because the effectiveness of different ML processes has been proven to vary depending on the kind of data or applicant (20).

As a result, the goal of this research was to investigate effective radiomics-based ML algorithms that predict ALK rearrangements in patients with NSCLC.

## Materials and methods

### Patients inclusion

**Figure 1:**
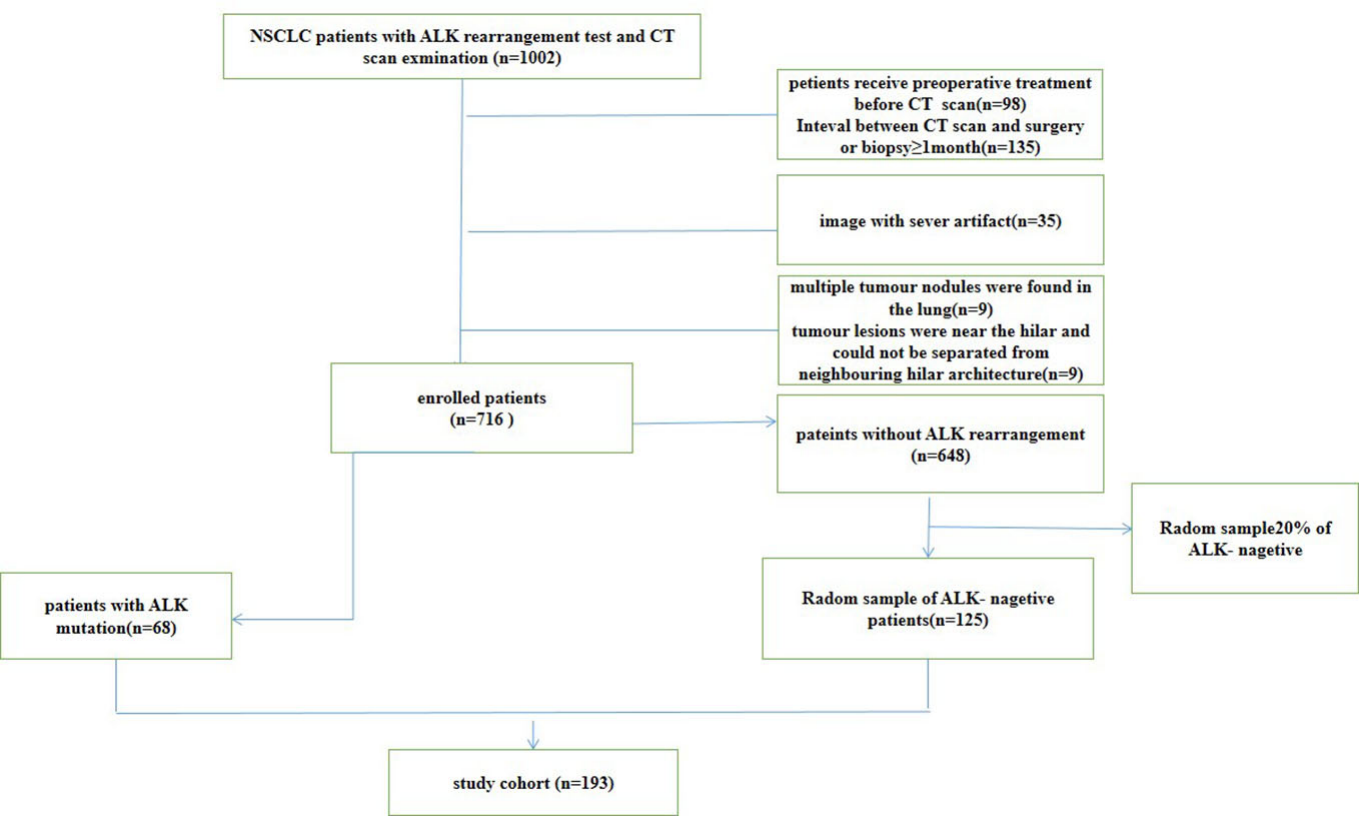
The flowchart of patients selection for non-small cell lung cancer (NSCLC).

From May 2012 to February 2020, we retrospectively reviewed all CT scans of NSCLC patients from PACS system at Nanfang hospital. This retrospective study examined 1002 patients with pathologically proven lung cancer who underwent surgery or received a biopsy. The clinical features of the patients were retrieved from the hospital information system. This study included patients over the age of 18 who met the following criteria: (1) had tumour specimens with confirmed ALK gene rearrangements and pathological testing; (2) had pretreatment CT images; and (3) had complete clinical data. The exclusion criteria were as follows: (1) patients receiving treatment before CT scan (2) the time between CT examination and treatment was longer than one month; (3) multiple tumour nodules were found in the lung; (4) tumour lesions were near the hilar and could not be separated from neighbouring hilar architecture. According to these criteria, 716 patients were included, 648 of whom were ALK negative and 68 of whom were positive for ALK rearrangements. Twenty percent of the ALK negative patients were randomly chosen to participate in our study. Finally, this study included 193 patients, 125 ALK negative patients and 68 ALK positive patients. The flowchart of patients selection for non-small cell lung cancer (NSCLC) was show in **Figure 1**. The TNM system was utilized for staging based on the American Joint Committee on Cancer (AJCC) manual (21). The patients were divided into two groups: a primary cohort (n=154 patients) and an independent validation cohort (n=39 patients), were randomly chosen in a ratio of 8:2 from patients with and without ALK rearrangements. This study was approved by the Ethical Committee of the Nanfang Hospital.

The patients were examined with 256-slice iCT (Philips Health care, Best, Netherlands) or Siemens Medical Solutions’ Sensation 64, Definition AS (Forchheim, Germany) equipment. The scanning parameters of the two scanners were as follows: tube rotation time 0.5 s vs. 0.5 s, pitch 0.87 vs. 1.2, detector collimation 128 0.625 vs. 64, tube voltage 120 kV, tube current 100-300 mA, field view 350 mm, matrix 512×512, slice thickness 1-5 mm, reconstruction interval 1 mm, and voxel spacing (X and Y directions) 0.52-1.36 mm. Two different scanners from different manufacturers were adopted. Standardization and normalization were applied to all matrices before analysis (22).

### Analysis of ALK rearrangement status

For genetic status determination, tissue samples acquired from biopsy or surgical excision were employed. The tissue specimens were prepared using formalin fixation and paraffin embedding. Immunohistochemistry with the D5F3 antibody, which has already been widely utilized for this purpose, was employed to detect ALK rearrangement gene expression. Two senior pathologists validated the findings. Wild-type ALK was defined as a specimen that did not have the ALK fusion gene present.

### Radiomic analysis

**Figure 2:**
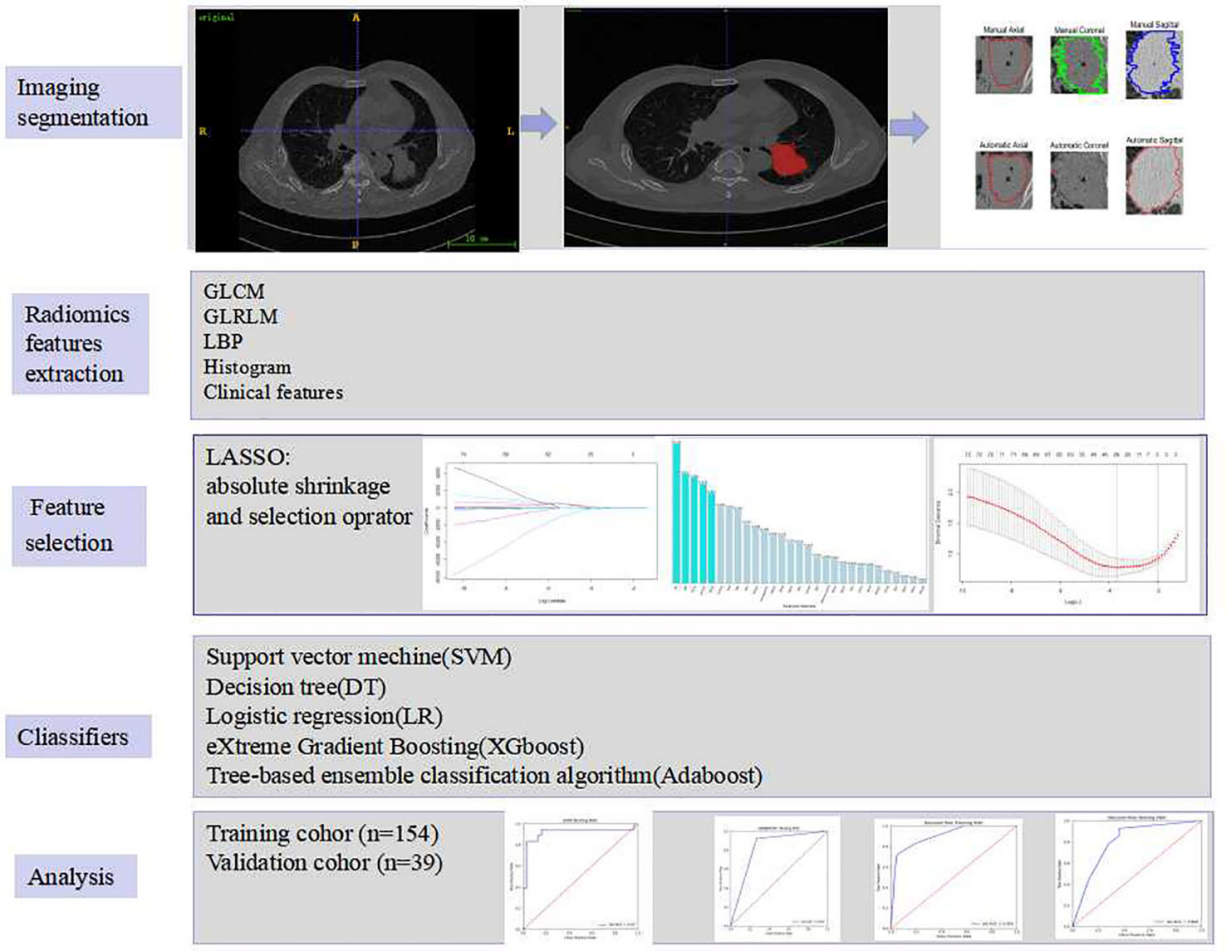
The radiogenomics framework of this study.

The radiomic analysis included five steps, which was illustrated in **Figure 2**.

### Segmentation of tumours

For each patient, horizontal, coronal and sagittal views were used for tumour segmentation by using ITK-SNAP software (http://www.itksnap.org/). A chest radiologist with 8 years of experience manually segregated the regions of interest (ROIs) and a senior with ten years of expertise reviewed all the ROIs. To ensure segmentation reproducibility, 30 patients were chosen at random to validate the inter-observer agreement between two observers’ delineations of ROIs using the dice similarity coefficient (DSC) by using Matlab 2018b, average value:0.8349 (from 0.6680 to 0.9186).To illustrate,the level of volume agreement the categorization scale below was used: DSC≥0.85 (High Agreement), 0.85>DSC≥0.70(Medium Agreement), 0.7>DSC≥0.5 (Low Agreement), DSC<0.5(Very Low Agreement) (23) Some inappropriate segmentations for ROI bounds were modified where necessary. An automatic active contour segmentation method was used to refine the manually segmented findings.

### Radiomic features extraction

Radiomic characteristics were extracted from two-dimensional region of interest (2D-ROI). Pyradiomics (http://pyradiomics.readthedocs.io/en/latest/index.html) was utilized. To be potentially clinically beneficial, we constructed classifiers based on radiomic features acquired from each ROI. Grey level co-occurrence matrix (GLCM), grey level run length matrix (GLRLM), local binary pattern (LBP), histogram, and clinical parameters were acquired from each ROI. The texture and clinical features were then normalized.

### Feature selection and classification algorithms

The LASSO technique and 10-fold cross-validation were used to obtain the best subset of radiomic characteristics. A variety of classifiers, including support vector machine (SVM), eXtreme gradient boosting (XGboost), tree-based ensemble classification algorithm (Adaboost), decision tree (DT) and logistic regression (LR), were assessed. The model’s performance was evaluated using receiver operating characteristic (ROC) curves and the area under the ROC curve (AUC) by 100 repeated tests. Accuracy, sensitivity, F1, recall and mean absolute error were all calculated as well.

### Statistics analysis

Data was analysed with IBM SPSS 25.0 (http://www.ibm.com). For continuous variables, the two independent samples t-test or the Mann–Whitney U test were used, and the significant differences in the ML model between the ALK^+^ and ALK^-^ groups were investigated using the same statistical methodologies. For categorical variables, such as gender, history of smoking, smoking index, clinical stage, distant metastasis, and tumour’s degree of pathological invasiveness and EGFR mutation of tumour, the chi-square test or Fisher’s exact test was used. The difference in AUCs between the two models was calculated statistically using DeLong’s test. The ML model was implemented using the Keras toolkit and Python (version 3.6.8, https://www.python.org/).

## Results

### Patient Cohort

The clinical characteristics of the patients were described in **Table 1**. The ALK rearrangement-positive patients were significantly younger than the ALK rearrangement-negative individuals (*P* < 0.001). In addition, more patients with stage III-IV cancer were found in the ALK mutation group (*P* < 0.001).

**Table 1:** Clinical characteristics of the ALK (+) and ALK (-) patients.

Characteristics	ALK (+)	ALK (-)	P-value^b^
	(n = 68)	(n = 125)	
Age (years^a^)	50.94 ± 12	57.57 ± 10.3	<0.001*
Gender			0.748
Males	31 (46)	60 (48)	
Females	37 (54)	65 (52)	
Smoking status Never Former Current	52 (76) 10 (15) 6 (9)	92 (74) 24 (19) 9 (7)	0.704
SI (pack-years) SI ≤ 10 10 < SI < 20 SI ≥ 20	52 (76) 6 (9) 10 (15)	85 (68) 22 (18) 18 (14)	0.248
EGFR mutation Positive Negative	0 (0) 68 (100)	74 (59) 51 (41)	<0.001*
Pathology features AIS IVC	1 (1) 67 (99)	9 (7) 116 (93)	0.169
TNM stage I-II III-IV	7 (10) 61 (90)	57 (46) 68 (54)	<0.001*
DM Positive Negative	55 (81) 13 (19)	62 (49.6) 63 (50.4)	<0.001*

a Mean ± standard deviation (range).

b ALK– group vs. ALK+ group.

*P < 0.05.

ALK, anaplastic lymphoma kinase; AIS, adenocarcinoma *in situ*; IAC, invasive adenocarcinoma; SI, smoking index; DM, distant metastasis.

### Performance of the radiomic machine learning algorithm

**Figure 3:**
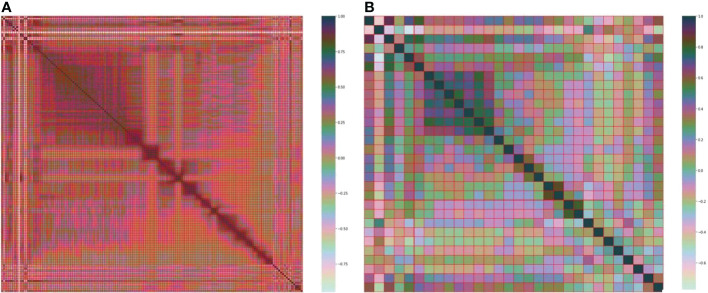
**(A)** Heatmap of the 157 radiomic features. **(B)** Heatmap of the 30 most important radiomic characteristics.

**Figure 4:**
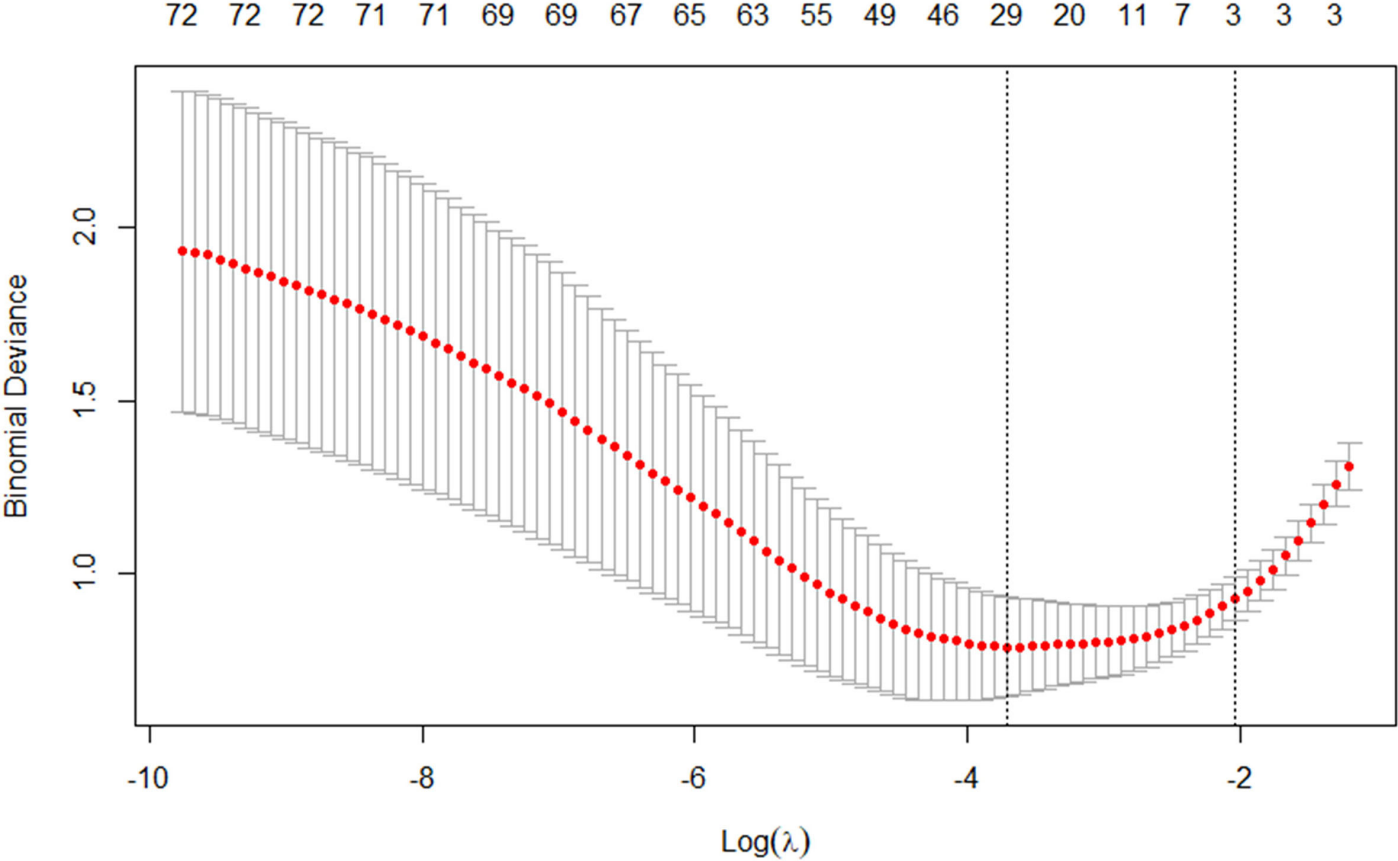
The tuning parameter in the LASSO model was chosen using a 10-fold cross-validation method based on minimum criterion. The LASSO regression cross-validation model’s binomial deviances as a function of logs(λ) were plotted.

**Figure 5:**
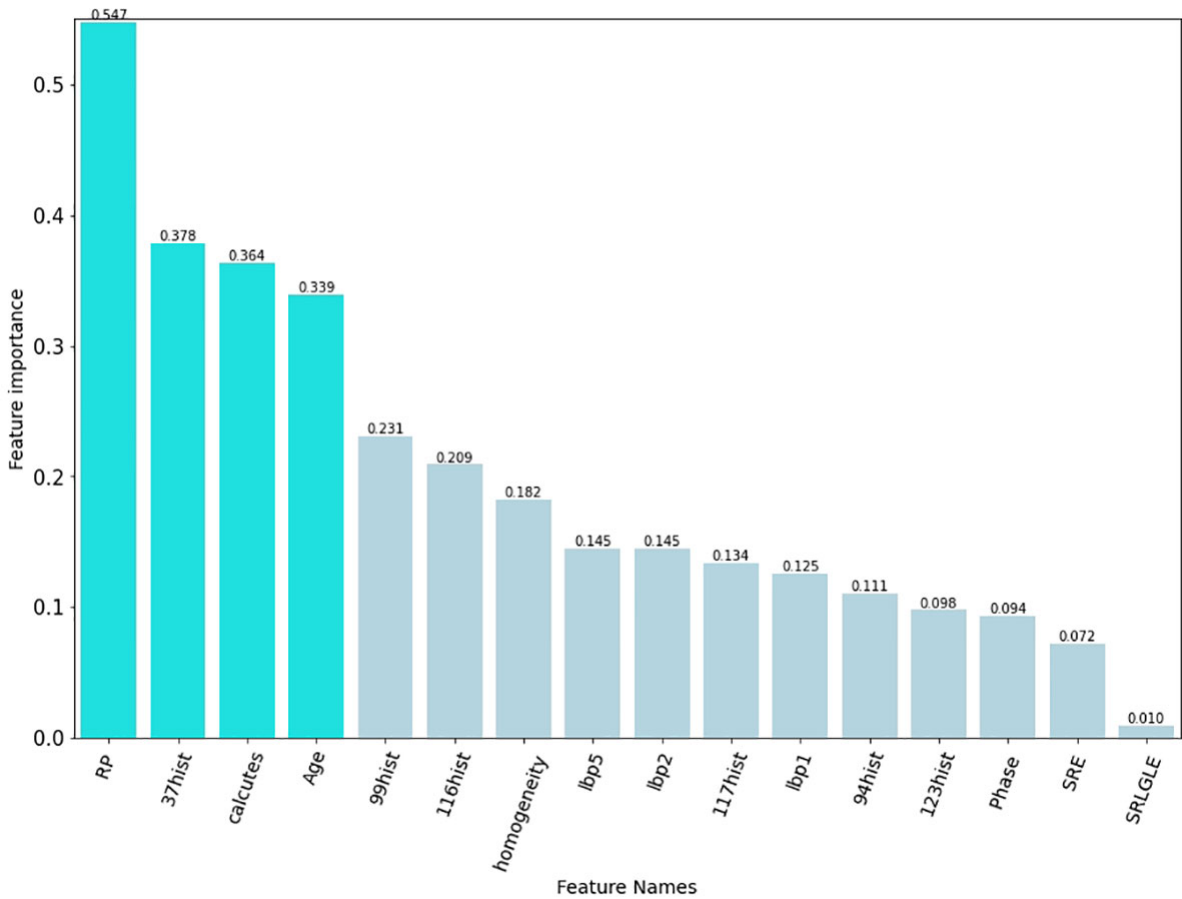
The final elements that were chosen to be maintained. The preserved characteristics were on the y axis, and the matching LASSO regression coefficients were on the x axis. The log(λ) coefficients of the features that have been fitted.

The best subset of radiomic characteristics was selected using the LASSO technique and 10-fold cross-validation. The radiomic features were retrieved from the 193 patients in the training set to create the radiomic signature. (**Figures 3**–**5**). The chosen radiomic properties were as follows: 37hist, 94hist, 99hist, 116hist, 117hist, 123hist, calcutes, homogeneity, Lbp1, Lbp2, Lbp5, PR, SRE, SALGLE (**Table 2**). Age and phase are proved significant clinical features.

**Table 2:** Selected features with descriptions.

Feature Name	Description
Gray-level co-occurrence matrix (**GLCM**) Homogeneitycalutes	Localization of regions with significant intensity changes; gradients detect edges and quantify region boundaries
Gray-level run length matrix (**GLRLM**) Short-run emphasis (SRE)Short-Run Low Gray-Level Emphasis (SRLGLE) Run percentage (RP)	Measure of the gray scale texture repeatability
Local binary pattern (**LBP**) Lbp1 Lbp2 Lbp5	The lbp (local binary pattern) is an operator used to describe the local texture features of an image.Reflects the content of each pixel to the surrounding pixels.
**Histogram** 37hist 99hist 94hist 116hist 117hist 123hist	Refect the distribution of voxel gray intensity

### Supervised learning classification

**Figure 6:**
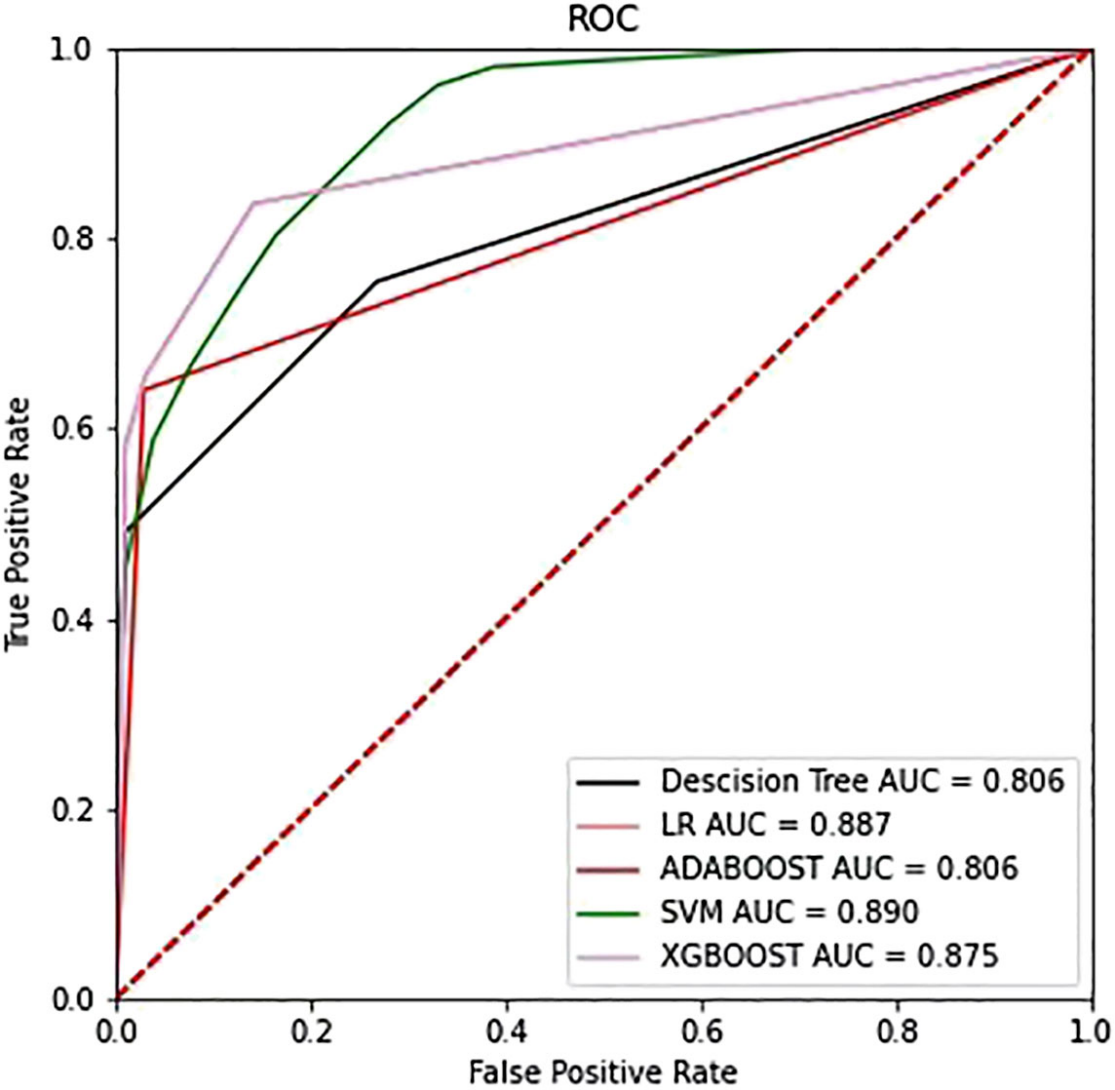
The ROC curves of the top four models selected from the training phase on the testing dataset.

After applying SVM, XGboost, Adaboost, LBP, DT, and LR to determine the optimal features, we identified the most appropriate approach for generating the final classification model based on their performances. We also used grid-search cross-validation to find the best parameters for all of the ML techniques discussed above. In terms of detecting ALK mutations, SVM exceeded the other traditional ML methods as shown in **Tables 3**, **4** and **Figure 6**.

**Table 3:** Assessment of different classifier feature selection-based machine learning models for predicting ALK fusion type in the validation cohort.

	Accuracy	Precision	AUC	F1	Mean absolute error	Recall
LR	0.869	0.871	0.887	0.803	0.131	0.762
Adaboost	0.808	0.714	0.806	0.722	0.192	0.747
Decision Tree	0.812	0.790	0.806	0.708	0.188	0.665
XGBoost	0.842	0.822	0.875	0.759	0.158	0.720
SVM	0.849	0.932	0.890	0.747	0.151	0.63

**Table 4:** Assessment of different classifier feature selection-based machine learning models for predicting ALK fusion type in the training cohort.

	Accuracy	Precision	AUC	F1	Mean absolute error	Recall
LR	0.928	0.955	0.958	0.889	0.072	0.835
Adaboost	1	1	1	1	0	1
Decision Tree	0.894	0.913	0.906	0.836	0.106	0.778
XGBoost	0.989	0.990	0.996	0.983	0.011	0.977
SVM	0.943	0.985	0.953	0.911	0.057	0.851

### Predictive performance of the machine learning model

**Figure 7:**
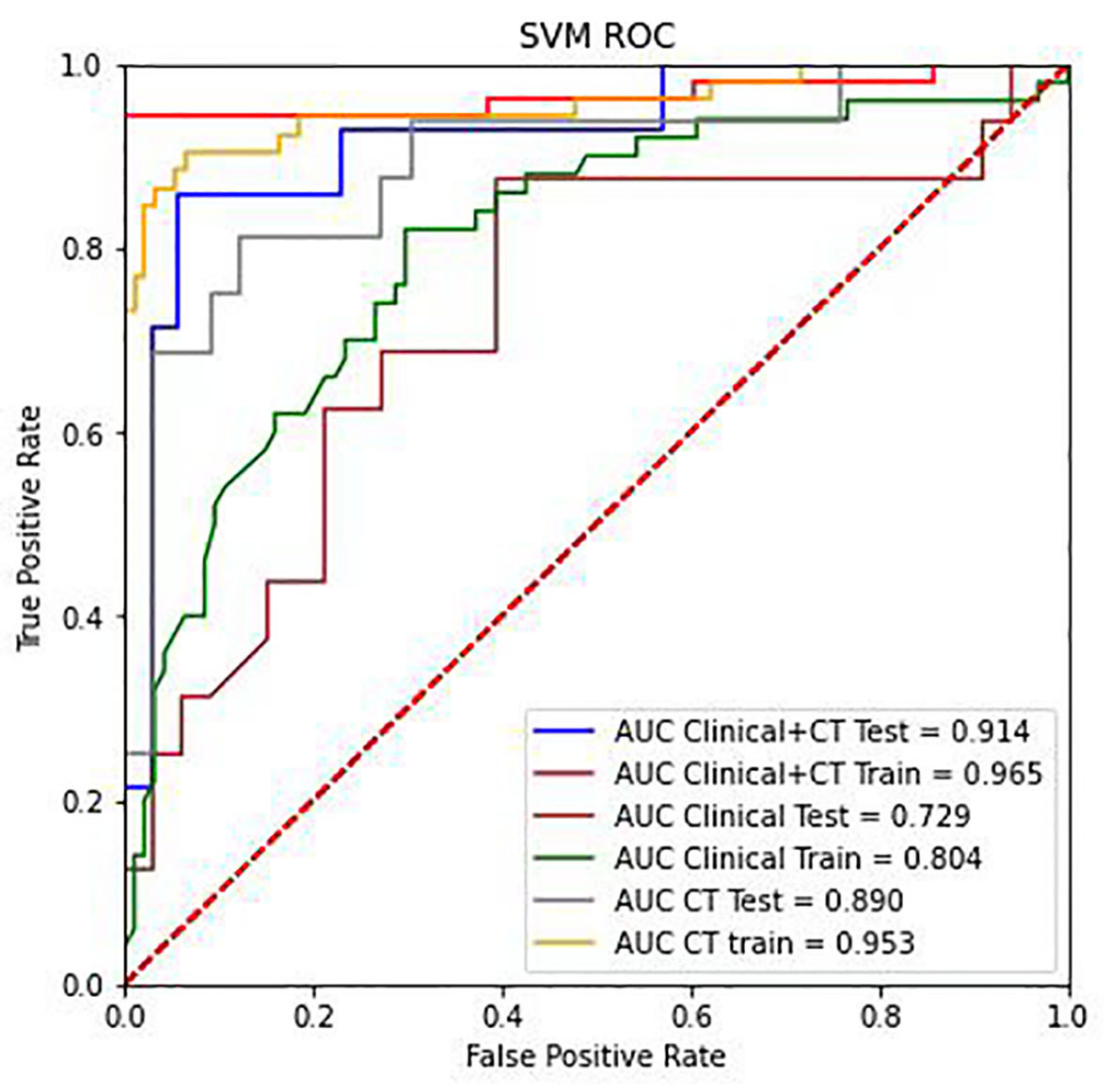
The ROC curve analysis of the CT radiomics models, clinical features, and combinations of CT radiomics and clinical features in the training group and testing group.

The SVM classifier had the highest AUC for classification (**Table 5**). In the primary cohort, the ML model trained on both CT scans and clinical features performed well AUC=0.965 (95% CI 0.8257–0.8823), which was verified in the validation cohort AUC=0.914 (95% CI 0.804–0.891; *P*<0.001). For the CT image-based model, the AUC was 0.953 (95% CI 0.913–1.0) and 0.890 (95% CI 0.778–0.971) for the primary and validation cohorts, respectively. The performance of the ML models trained on both CT images and clinical characteristics was significantly higher than that of the clinical model. The result for the primary cohort trained on the clinical model was an AUC=0.805 (95% CI 0.731–0.877; *P*<0.0001), and that for the validation cohort was an AUC=0.735 (95% CI 0.566–0.863; *P*<0.005). The decision curves are shown in **Figure 7**. The results indicated that ML models trained on both CT images and clinical data performed better than ML models trained by only CT images or the clinical characteristics.

**Table 5:** Predictive performance of SVM in the primary and validation cohorts.

Model	Cohorts	Accuracy	Precision	AUC	F1	Mean absolute error	Recall
Clinacal features	Primary	0.758	0.826	0.805	0.612	0.242	0.569
	Validation	0.689	0.5	0.735	0.483	0.311	0.455
CT image	Primary	0.923	0.946	0.953	0.877	0.077	0.792
	Validation	0.858	0.938	0.890	0.769	0.142	0.675
CT image and clinigal features	Primary	0.943	0.985	0.965	0.911	0.057	0.851
	Validation	0.849	0.932	0.914	0.747	0.151	0.63

## Discussion

Our findings demonstrated that using the SVM classifier to predict ALK gene rearrangements based on both CT scans and clinical characteristics was the most effective strategy. In our study, the integrated model exhibited the highest AUC, which exceeds the clinical models based on previously identified CT characteristics (also known as morphological or semantic CT features) (12, 24) and combined with clinical features, semantic CT features, and radiomic features (25).

Among clinical features, we found ALK^+^ patients are often younger than ALK^-^ patients, which is consistent with prior studies (7). Female sex and smoking history, on the other hand, do not differ much between the two groups of patients. The bulk of the patients in our ALK^+^ study was in advanced stages of cancer (III-IV). ALK gene rearrangements were more common in lung cancer at advanced stages, in accordance with a prior study (10). Clinical information is useful in improving the integrated model for ALK rearrangement status detection, as it increases the integrated model’s performance by incorporating clinical characteristics for lesions in the primary and validation cohorts. A previous study had proposed a predictive model for detecting ALK rearrangements, using age as the only clinical component selected and many semantic CT features (AUC=0.846) (9).

Our findings imply that radiomics can be used to predict ALK rearrangement status on CT images in patients with NSCLC. The histogram and texture categories served as the foundation for the radiomic model, which implies that the intensity change of tumours was a potent predictor of the ALK genetic mutation. In this study, we found that Rp, 37hist, and calutes, that linked with ALK mutations. The AUC for postcontrast CT radiomic characteristics detecting ALK rearrangements was 0.829, according to Ma et al. (26). However, their research was based on enhanced CT scans. The radiomic model in our study demonstrates that radiomic features extracted from nonenhanced CT images are sufficient for developing a reliable ALK rearrangements prediction model in NSCLC patients.

Radiomics is an emerging discipline attempting to bridge the gap between medical imaging and personalized medicine (27, 28) by investigating the value of medical images in the diagnosis, grading, and prognostication of diseases using medical image analysis technologies and ML algorithms. However, the best way to use certain medical images or objectives is unclear due to the various feature selection approaches and ML algorithms (29).

In recent years, researchers have investigated the efficacy of various feature selection and ML algorithms in medical image classification to determine whether they are appropriate for the given medical image data. For example, Shiri I et al. (20) examined radiomic characteristics from low-dose CT, diagnostic quality CT, and PET-CT as well as ML techniques in NSCLC patients. Their results predicted mutation status of EGFR and KRAS. Then, six feature selection procedures and 12 classifiers were used, and multivariate ML-based AUC performances for EGFR and KRAS were improved to 0.82 and 0.83, respectively. Lan Song et al. compared the performance of three feature selection approaches and two classification methods for predicting ALK fusion in lung cancer patients using clinical characteristics combined with conventional CT and radiomic data (25). They extracted 1218 radiomic characteristics from CT scans and discovered that the LR and DT classifiers had the best prediction performance (AUC=0.890). The optimal ML classifier and feature selection method varied between studies, which could be related to a variety of factors, such as visual modalities, feature extraction algorithms, the number of features chosen, the goal task, and cohort size. According to Han’s study, radiomics-based ML was used to determine the best model for NSCLC histologic subtypes (29), and SVM paired with LASSO produced the highest prediction efficacy, similar to our study.

Even though our model’s performance was quite promising, there are a few limitations in this study that need to be addressed. First, although the results were favourable, the model’s ability to handle imbalanced data must be improved to generalize the prediction outcome to more datasets. Second, we may need to employ a cutting-edge deep learning method to perform the classification task is warranted. Several studies have successfully constructed models to address this issue with positive results (30). These findings have encouraged us to use neural networks to construct the baseline model in future studies.

In conclusion, the ML model that combined CT scans and clinical features are able to accurately identify the status of the ALK gene. This study provides a noninvasive solution, which is a quick and simple way to guide clinical genetic diagnosis.

## Data availability statement

The original contributions presented in the study are included in the article/supplementary material. Further inquiries can be directed to the corresponding author.

## Ethics statement

The studies involving human participants were reviewed and approved by The Nanfang Hospital’s Ethical Committee. The patients/participants provided their written informed consent to participate in this study. Written informed consent was obtained from the individual(s) for the publication of any potentially identifiable images or data included in this article.

## Author contributions

Guarantor of integrity of the entire study: PH, B-YD, WZ and Y-KX. Study concepts and design: PH, Z-XL and Y-KX. Literature research: PH, B-YD. Clinical studies: JX, C-TH, FZ. Experimental studies/data analysis: B-YD, WZ. Statistical analysis: FZ. Manuscript preparation: PH, Z-XL and Y-KX. Manuscript editing: PH, B-YD. PH and B-YD contribute equally to this work. All authors contributed to the article and approved the submitted version.

## Funding

Natural Science Foundation of Guangdong Province (NO. 2017A030310102).

## Acknowledgments

We thank the SPRINGER NATURE Author Services for its linguistic assistance during the preparation of this manuscript.

## Conflict of interest

The authors declare that the research was conducted in the absence of any commercial or financial relationships that could be construed as a potential conflict of interest.

## Publisher’s note

All claims expressed in this article are solely those of the authors and do not necessarily represent those of their affiliated organizations, or those of the publisher, the editors and the reviewers. Any product that may be evaluated in this article, or claim that may be made by its manufacturer, is not guaranteed or endorsed by the publisher.
